# Association between craniofacial anomalies, intellectual disability and autism spectrum disorder: Western Australian population-based study

**DOI:** 10.1038/s41390-022-02024-9

**Published:** 2022-03-29

**Authors:** Mohammed Junaid, Linda Slack-Smith, Kingsley Wong, Jenny Bourke, Gareth Baynam, Hanny Calache, Helen Leonard

**Affiliations:** 1 School of Population and Global Health, The University of Western Australia, Clifton Street Building, Clifton Street, Nedlands, 6009 WA Australia; 2 Telethon Kids Institute, The University of Western Australia, Northern Entrance, 15 Hospital Avenue, Nedlands, 6009 WA Australia; 3 Western Australian Register of Developmental Anomalies, Department of Health, Government of Western Australia, Perth, WA Australia; 4 Deakin Health Economics, Institute of Health Transformation, School of Health and Social Development, Faculty of Health, Deakin University, Geelong, VIC Australia; 5 Department of Dentistry and Oral Health, La Trobe Rural Health School, La Trobe University, Bendigo, VIC Australia

## Abstract

**Background:**

Accurate knowledge of the relationship between craniofacial anomalies (CFA), intellectual disability (ID) and autism spectrum disorder (ASD) is essential to improve services and outcomes. The aim is to describe the association between CFA, ID and ASD using linked population data.

**Methods:**

All births (1983–2005; *n* = 566,225) including CFA births (comprising orofacial clefts, craniosynostosis, craniofacial microsomia and mandibulofacial dysostosis) surviving to 5 years were identified from the birth, death, birth defects and midwives population data sets. Linked data from these data sets were followed for a minimum of 5 years from birth until 2010 in the intellectual disability database to identify ID and ASD. These associations were examined using a modified Poisson regression.

**Results:**

Prevalence of ID and ASD was higher among CFA (especially with additional anomalies) than those without [prevalence ratio 5.27, 95% CI 4.44, 6.25]. It was higher among CFA than those with other gastrointestinal and urogenital anomalies but lower than nervous system and chromosomal anomalies. Children with CFA and severe ID had a higher proportion of nervous system anomalies.

**Conclusions:**

Findings indicate increased ID and ASD among CFA but lower than nervous system and chromosomal anomalies. This population evidence can improve early identification of ID/ASD among CFA and support service planning.

**Impact:**

- Our study found about one in ten children born with craniofacial anomalies (CFA) are later identified with intellectual disability (ID).
- Prevalence of ID among CFA was higher than those with other gastrointestinal, urogenital, and musculoskeletal birth defects but lower than those with the nervous system and chromosomal abnormalities.
- Most children with craniofacial anomalies have a mild-to-moderate intellectual disability with an unknown aetiology.
- On average, intellectual disability is identified 2 years later for children born with non-syndromic craniofacial anomalies than those with syndromic conditions.
- Our findings can improve the early identification of ID/ASD among CFA and support service planning.

## Background

Craniofacial anomalies (CFA) are a heterogeneous group of structural birth defects resulting either from disturbances in craniofacial development or as a secondary feature to other congenital conditions (multiple congenital anomalies).^1,2^ CFA can be classified based on aetiological criteria as disruptions in normal development (oral clefts), deformations from extrinsic mechanical compression (amniotic band sequence), malformation sequences (Pierre Robin sequence), chromosomal abnormalities, monogenic disorders (Van der Woude syndrome), dysostosis (craniosynostosis (CS), mandibulofacial and acrofacial dysostosis) and osteochondrodysplasias.^3^ Affected individuals encounter many challenges apart from structural defects, including feeding, hearing and speech difficulties that require multidisciplinary care often extending until early adulthood.^4–6^ These repeated episodes of hospitalisations may be associated with emotional disturbances and social isolation leading to high absences from school and workplace exclusion.^7–9^

In addition to physical health, cognitive development is an area of concern. This stems from the likelihood that facial malformations (especially clefting) may be associated with aberrant brain development as both are derived from common ectodermal tissues and their development is closely linked during early morphogenesis.^10,11^ Furthermore, some CFA especially oral clefts and craniofacial microsomia (CFM) have already been found to be associated with a lower verbal intelligence quotient (IQ), learning disabilities, poor academic achievement, deficits in verbal fluency and rapid verbal labelling, short-term memory and with an increased risk of autism spectrum disorder (ASD; cleft lip and palate only).^12–16^ On the other hand, most children with CFA especially non-syndromic oral clefts and CS, Treacher Collins syndrome and CFM, have demonstrated normal intelligence when measured by full-scale IQs.^17–20^ However, there is a paucity of relevant population-level data.

Intellectual disability (ID) is “characterised by significant limitations both in intellectual functioning and in adaptive behaviour as expressed in conceptual, social and adaptive skills” and originates before the age of 18 years.^21^ ASD refers to a group of neurodevelopmental disorders characterised by qualitative impairments in social interaction, communication with restricted repetitive behaviour.^22^ While the prevalence of ID and ASD vary widely depending on the methodology of studies globally, the prevalence of ID and ASD in Western Australia (WA) has been estimated at 17 per 1000 live births and 5.1 in 1000 live births.^23–25^

Both ID and ASD are more common among children born with a birth defect; however, the diagnosis of ID and ASD often occurs after infancy.^26,27^ Furthermore, some maternal antenatal aetiological risk factors including maternal epilepsy, diabetes and advanced maternal age have been associated with CFA and ID separately but not synonymously.^28,29^

Considering CFA is comprised of a heterogeneous group of disorders including rare conditions,^2^ an accurate knowledge of their relationship with ID and ASD could assist with earlier identification^26^ which may improve planning of subsequent treatment and individual outcomes.

Hence, the aims of this Western Australian linked total population data study were thus twofold. First, to determine the prevalence of ID and ASD among individuals with CFA compared to those born without. Second, to explore the associations between ID and CFA and their relationship with demographic, antenatal and perinatal factors using linked total population data for WA.

## Methods

### Data sources

In this retrospective cohort study, all births (1983–2005) including CFA surviving to 5 years were identified from four different administrative data sets and followed for a minimum of 5 years from birth until 2010 in the Intellectual Disability Exploring Answers (IDEA) database to ascertain the prevalence of ID/ASD. This study utilised record-linked de-identified data from the total of five above-mentioned administrative data sets made available by the Western Australian Data Linkage System (WADLS). The data sets used in this study included: the Western Australian Register of Developmental Anomalies—Birth Defects (WARDA-BD), the Midwives Notification System (MNS), Western Australian Birth and Death Registrations and the IDEA database (Supplementary Fig. S1).

Cases of CFA and other congenital anomalies were identified from WARDA-BD, a population-based statutory register of congenital anomalies with multiple sources of ascertainment of structural and functional anomalies diagnosed in live births up to six years of age as well as in stillbirths. Each individual congenital anomaly is coded using the British Paediatric Association International Classification of Diseases Ninth revision system (BPA-ICD9), allowing provision for 10 diagnostic categories to code for multiple anomalies per infant.^30^ Information related to the Indigenous status, antenatal and perinatal factors were extracted from the MNS which is a legislated surveillance system that collects information on all births attended by midwives in WA with the gestational age of ≥20 weeks or birth weight ≥400 g.^31^ The Western Australian Birth Registrations contains all registered births and also provides information related to parental age.^32^ The Western Australian Death Registrations provides information on all registered deaths and cause of mortality.^33^

Children with ID were ascertained using the IDEA database.^34^ Cases with ID are currently identified through the Disability Services Commission (now Department of Communities) and the Education Department of WA using age-appropriate assessment tools such as Wechsler intelligence scales.^35^ The Disability Services Commission (DSC) ascertains ID if general intelligence among eligible individuals is estimated to be more than two standard deviations (SDs) below the mean for their age (or full-scale IQ < 70) and if the individual has proportionate deficits in adaptive behaviours manifesting before 18 years of age or they were diagnosed with a condition which is consistent with ID (e.g. Down syndrome).^35,36^ For younger children aged up to about 6 years a relevant development test such as the Griffiths Scales may be used for assessment and scores <70 may be described as vulnerable for ID.^36^ Children with ASD were also ascertained through DSC and diagnosed using either the Diagnostic and Statistical Manual of Mental Disorders (DSM) Fourth or Fifth edition criteria [DSM-IV or DSM-V].^22^ Evidence of comorbid ID is recorded where available.

The WADLS uses rigorous internationally accepted privacy-sensitive protocols along with computerised probabilistic matching and extensive clerical review to link records for an individual occurring in many different administrative data collections in WA.^37^ As a result of this linkage, each individual record is provided with a common unique identification number (linkage key) which was used to merge WARDA-BD, MNS, birth, death registry and IDEA data sets to facilitate the analysis.

### Study population

The cases for this study comprised those born with CFA in WA between 1983 and 2005 surviving to 5 years of age. CFA for our study cohort were defined as major birth defects arising as primary disturbances in craniofacial development and categorised into four groups for analysis purposes: (i) orofacial clefts [includes cleft lip only, cleft palate only, cleft lip and palate and facial clefts] categorised as non-syndromic (identified as a single entity) and syndromic (with additional associated anomalies) oral clefts (ii) CS [includes non-syndromic (single suture synostosis) and syndromic synostosis], (iii) CFM was defined as the presence of microtia/ear anomalies and/or at least one major anomaly of the oculo-auricular vertebral spectrum.^38^ Goldenhar syndrome is identified as a severe variant of CFM with vertebral involvement,^38^ (iv) mandibulofacial dysostosis specifically Treacher Collins syndrome.

The CFA were identified by BPA-ICD9 CM codes with diagnostic descriptions of cleft palate only (74900–74909), cleft lip only (74910–74919), cleft lip and palate (74920–74929), facial cleft (74928), CS (75600), Crouzon syndrome (75601), Apert syndrome (75550), Pfeiffer syndrome (75601), Carpenter syndrome (75984), Saethre–Chotzen syndrome (75550), Muenke syndrome (75600), Baller-Gerold syndrome (75600), CFM (75400), facio-auricular vertebral syndrome/Goldenhar syndrome (75606), Pierre Robin syndrome (75603), Treacher Collins syndrome (75604) and Van der Woude syndrome (75980). Cases of oral clefts and CS without any associated anomalies were defined as non-syndromic.

The case group excluded individuals born with multiple congenital anomalies (where CFA were not a major disease finding), isolated non-syndromic tongue disorders, salivary gland disorders, dental abnormalities, nasal, optic and otic abnormalities.

### Comparison cohort

The comparison cohort (*n* = 564,804) included all children surviving to 5 years of age over the same period but not diagnosed with CFA. This was extracted from the MNS. We also created a sub-group from within the existing comparison cohort comprising of children surviving to 5 years born with other congenital anomalies (*n* = 28,632) besides CFA, identified using BPA-ICD9 CM codes from WARDA-BD. These congenital anomalies were further categorised by system as follows: nervous (74000–74299), cardiovascular (74500–74799), respiratory (74800–74899), gastrointestinal (74900–75199), urogenital (75200–75899), musculoskeletal (75400–75699) and integument (75710–75792). Apart from system categories, we also identified chromosomal diagnoses (75800–75899) which included autosomal and allosomal chromosomal defects.

### Outcome

The main outcomes in this study were ID and ASD. The level of ID was grouped as mild/moderate (IQ 69-40) or severe (IQ < 40) because eligibility identified by the education system does not allow differentiation between mild and moderate levels of ID.^36^ ID was also grouped based on aetiology and level of disability as (1) ID with co-morbid autism, (2) known biomedical case (such as genetic disorders, teratogenic conditions, foetal alcohol syndrome, chromosomal disorders or postnatal injury), (3) unknown cause with mild or moderate ID, (4) unknown cause with severe ID.^39^ Cases with ID are labelled as unknown aetiology if no definite cause has been identified or if cases have only been ascertained by education sources alone without relevant medical information.^36^ In this study, ASD was grouped from categories (1) ASD without ID (2) ASD with concurrent ID as identified in the IDEA database.

### Covariates

#### Demographic factors

Information related to infant sex, parental age, birth year and Indigenous status for case and comparison cohorts was collected from the WA Birth Registrations. Postcodes of residence at birth were used to categorise remoteness of geographic location utilising the Accessibility and Remoteness Index of Australia (ARIA) for 2006 made available in the MNS. ARIA is a unique indicator of remoteness and ‘rurality’ and is relatively stable over time.^40^ Similarly, collection districts relating to mother’s residence at the time of her infant’s birth were categorised into socio-economic quintiles based on their Socio-Economic Indexes for Areas (SEIFA) specifically utilising the Index of socioeconomic Disadvantage (IRSD) from respective census years, according to the year of birth. This index (IRSD) summarises measures of relative disadvantage based on socio-economic conditions of people and households within an area. A low score indicates greater disadvantage (households with low income, people with no qualifications or in low skill occupations) and a higher score would indicate relative lack of disadvantage.^41^

#### Antenatal factors

We extracted data related to pre-existing maternal medical conditions, fertility treatments and smoking from the MNS. We investigated selected pre-existing maternal medical conditions such as diabetes mellitus, epilepsy and hypertension using the respective ICD-9-CM/ICD-10-AM codes (Supplementary Table S1). We considered mothers with pre-existing epilepsy as a proxy variable for use of anti-seizure medications. Binary data related to fertility treatments and smoking (active) has only been available since 1993 and 1997 respectively.

#### Perinatal factors

Perinatal variables from the MNS included parity, plurality, gestational age, birth weight, 5-min Apgar score, foetal distress, resuscitation, presentation of foetus and mode of delivery. Among other perinatal factors assessed, intrauterine growth restriction was determined using the proportion of optimal birth weight, which refers to the ratio of observed birth weight to the (calculated) optimal birth weight.^42^ We also assessed the percentage of optimal head circumference, collected from 1990, defined as ratio of infant head circumference to the optimal head circumference.^43^

### Statistical analyses

Descriptive statistics, reported as median (interquartile range (IQR)) or count (%) where appropriate, were used to summarise the characteristics of the study population (e.g., ID by severity and cause, ASD, CFA by contributing diagnoses and syndromes, and age of diagnosis of ID and ASD).

Associations of ID with CFA, reported as prevalence ratios and their 95% confidence intervals (CIs), were estimated using the modified Poisson regression with the Huber-White sandwich estimator of variance. This method was used to overcome the issue of incorrect estimation of error when regular Poisson regression is used for binary data.^44^ Both unadjusted and adjusted regression analyses were performed. As had been previously done,^26^ the adjusted analysis included confounders such as sex, Indigenous status, ARIA, SEIFA (IRSD), maternal age, parity, plurality, gestational age and birth weight.

For the comparison group used in the analysis, we have chosen the predefined comparison cohort and, in addition, a subset of the cohort (*n* = 28,362) that only contained children with other congenital anomalies but CFA (Supplementary Fig. S2).

Missing values were found in a number of covariates, with 5% missing data in residence remoteness and socio-economic status (Supplementary Tables S2 and S3). Mechanism of missingness was assumed to be missing at random and complete case analysis was used. This approach was considered valid due to the probability of complete record was found not to be dependent on the outcome variable, given the exposure and covariates.^45^

To investigate the differential associations of demographic, antenatal and perinatal factors between ID and CFA, interaction terms involving these variables were constructed using the product term approach. These interaction terms were separately added to the regression models, with the selected confounders and resulting relative prevalence ratios were reported. We also reported the frequency distribution of the respective factors by ID and CFA status.

All analyses were carried out using Stata 16.0 (Stata Corp, College Station, TX).

### Ethics approval

The study protocol was approved by the Human Research Ethics Committee of the WA Department of Health (HREC#2011/64) and the University of WA (RA/4/20/5843).

## Results

The study flow diagram is shown in Supplementary Fig. S2. Of the 566,225 children born in WA between 1983 and 2005 and surviving to 5 years of age, 0.3% (*n* = 1421) were born with CFA, 1.7% (*n* = 9798) had ID and 0.4% (*n* = 2133) had ASD.

### Proportion of ID and ASD among CFA

Around 1 in 10 (9.4%, *n* = 134; 94.3 per 1000 live births) children diagnosed with CFA were identified with ID and 0.8% (*n* = 11; 7.74 per 1000 live births) were identified as having ASD with or without comorbid ID (Table 1). In the ID subgroup, the majority was categorised as mild or moderate (85.1%, 114/134) and a small proportion was diagnosed as severe cases (12.7%, 17/134). The number of children with CFA categorised as having an unknown cause of ID (*n* = 70) was higher than that number of children with CFA having a biomedical cause of ID (*n* = 57). The pattern of distribution of ID and ASD among CFA children was similar to that observed for other congenital anomalies but distinctively higher than the comparison cohort without CFA (Table 1). Among contributing diagnoses of CFA, syndromic orofacial clefts (19.8%) and syndromic CS (28.7%) were found to have a higher proportion of ID relative to non-syndromic orofacial clefts (2.7%) and non-syndromic CS (2.5%), respectively (Table 1). However, almost all cases of non-syndromic orofacial clefts (94%, 17/18) had an unknown cause of ID. We identified ID among Crouzon, Apert and Pfieffer syndrome (*n* < 5) but there was no ID in children with Muenke syndrome and Treacher Collins syndrome.

**Table 1:** Frequency and prevalence of intellectual disability by category of disorder and autism spectrum disorder among individuals born with craniofacial anomalies.

Craniofacial anomalies and subtypes (*n*)	Intellectual disability	Intellectual disability, by severity			Intellectual disability, by cause			Autism spectrum disorder^a^
		Mild or moderate	Severe	Unknown	Biomedical ID	Unknown mild–moderate	Unknown severe	
	*n* (%)	*n* (%)	*n* (%)	*n* (%)	*n* (%)	*n* (%)	*n* (%)	*n* (%)
*Craniofacial anomalies (1421)*	134 (9.4)^b^	114 (8.0)	17 (1.2)	≤5	57 (4.0)	65 (4.6)	≤5	11 (0.8)^c^
*Non-syndromic (878)*	23 (2.6)	23 (2.6)	0	0	≤5	20 (2.3)	0	≤5
*Syndromic (543)*	111 (20.4)	91 (16.8)	17 (3.1)	≤5	53 (9.9)	45 (8.4)	≤5 (0.9)	8 (1.5)
*Other congenital anomalies (28,362)*	2381 (8.4)	1952 (6.9)	361 (1.3)	68 (0.2)	1195 (4.2)	919 (3.2)	111 (0.4)	210 (0.7)
*Comparison cohort (564,804)*^d^	9664 (1.7)	8424 (1.5)	732 (0.1)	508 (0.1)	1556 (0.3)	6317 (1.1)	339 (0.001)	2122 (0.4)
*Contributing diagnosis*								
Orofacial clefts (1034)	89 (8.6)	76 (7.4)	10 (1.0)	≤5	33 (3.2)	47 (4.6)	≤5	6 (0.6)
Non-syndromic (673)	18 (2.7)	18 (2.7)	0	0	≤5	17 (2.5)	0	0 (0)
Syndromic (361)	71 (19.8)	58 (16.3)	10 (2.8)	≤5	32 (9.0)	30 (8.4)	≤5	6 (1.7)
Craniosynostosis (303)	34 (11.2)	28 (9.3)	≤5	≤5	17 (5.6)	13 (4.3)	≤5	≤5
Non-syndromic (202)	≤5	≤5	0	0	≤5	≤5	0	≤5
Sagittal synostosis (95)	≤5	≤5	0	0	≤5	≤5	0	0
Coronal synostosis (33)	≤5	≤5	0	0	0	≤5	0	≤5
Metopic synostosis (28)	0	0	0	0	0	0	0	≤5
Lambdoid synostosis (36)	0	0	0	0	0	0	0	≤5
Multiple suture synostosis (7)	0	0	0	0	0	0	0	0
Syndromic (101)	29 (28.7)	23 (23.0)	≤5	≤5	16 (16.0)	10 (10.0)	≤5	≤5
Craniofacial microsomia (78)	10 (12.8)	8 (10.5)	≤5	0	≤5	≤5	≤5	0
*Contributing syndromes*								
Crouzon syndrome (16)	≤5	≤5	0	0	≤5	≤5	0	0
Apert syndrome (≤5)	≤5	≤5	0	0	0	≤5	0	0
Pfieffer syndrome (≤5)	≤5	≤5	0	0	0	≤5	0	0
Muenke syndrome (≤5)	0	0	0	0	0	0	0	0
Baller Gerold syndrome (≤5)	≤5	≤5	0	0	≤5	0	0	0
Saethre–Chotzen syndrome (6)	≤5	≤5	0	0	≤5	0	0	0
Goldenhar syndrome (63)	9 (14.3)	7 (11.1)	≤5	0	≤5	≤5	≤5	0
Treacher Collins syndrome (≤5)	0	0	0	0	0	0	0	0
Pierre Robin sequence (106)	16 (15.1)	14 (13.2)	≤5	0	8 (8.0)	8 (8.0)	0	0
Van der Woude syndrome (13)	≤5	≤5	0	0	≤5	0	0	0

*n* number of individuals.

^a^Includes individuals with and without intellectual disability.

^b^Prevalence of intellectual disability among individuals with craniofacial anomalies (CFA): 94.3 per 1000 live CFA births.

^c^Prevalence of autism spectrum disorder among individuals with craniofacial anomalies (CFA): 7.74 per 1000 live CFA births.

^d^Includes individuals without craniofacial anomalies.

### Proportion of associated systemic anomalies among individuals with CFA and ID

Of the 134 children identified with both CFA and ID, nearly half were diagnosed with other additional musculoskeletal anomalies (48.5%) followed by anomalies of the nervous system (23.9%) (Table 2). In contrast for other congenital anomalies with ID there were higher proportions of cases diagnosed with chromosomal (30.6%) and cardiovascular anomalies (23.5%). The proportion of nervous system anomalies was even higher for CFA with severe ID (53%, 9/17) and included cases (*n* ≤ 5) of microcephaly, corpus callosum agenesis and holoprosencephaly. A similar pattern was observed for oral clefts, CS and CFM.

**Table 2:** Frequency and prevalence of associated congenital anomaly among individuals with craniofacial anomalies and intellectual disability.

Congenital anomalies diagnostic category (BPA-ICD9 code)	Craniofacial anomalies		Orofacial clefts		Craniosynostosis		Craniofacial microsomia		Other congenital anomalies	
	All ID (*n* = 134)	Severe ID (*n* = 17)	All ID (*n* = 89)	Severe ID (*n* = 11)	All ID (*n* = 34)	Severe ID (*n* ≤ 5)	All ID (*n* = 10)	Severe ID (*n* ≤ 5)	All ID (*n* = 2515)	Severe ID (*n* = 378)
	*n* (%)	*n* (%)	*n* (%)	*n* (%)	*n* (%)	*n* (%)	*n* (%)	*n* (%)	*n* (%)	*n* (%)
Chromosomal abnormalities (75800- 75899)	22 (16.4)	≤5	18 (20.2)	≤5	≤5	0	0	0	769 (30.6)	104 (27.5)
Foetal alcohol syndrome (75992)	≤5	0	≤5	0	≤5	0	0	0	47 (1.9)	6 (1.6)
System affected										
Nervous (74000–74299)	32 (23.9)^a^	9 (52.9)^b^	19 (21.4)	6 (54.6)	10 (29.4)	≤5	≤5	≤5	464 (18.5)	134 (35.5)
Cardiovascular (74500–74788)	22 (16.4)	≤5	15 (16.9)	≤5	≤5	0	≤5	≤5	591 (23.5)	65 (17.2)
Respiratory (74800–74888)	8 (6.0)	≤5	≤5	≤5	≤5	0	0	0	25 (1.0)	≤5
Gastrointestinal (75000–75188)	10 (7.5)	≤5	8 (9.0)	≤5	≤5	0	0	0	159 (6.3)	27 (7.1)
Urogenital (75200–75399)	24 (17.9)	≤5	17 (19.1)	≤5	6 (17.7)	0	0	0	498 (19.8)	49 (13.0)
Musculoskeletal (75400–75699)^c^	65 (48.5)	10 (58.8)	37 (41.6)	≤5	19 (55.9)	≤5	8 (80.0)	≤5	537 (21.4)	82 (21.7)
Integument (75710–75792)	8 (6.0)	≤5	≤5	0	≤5	≤5	≤5	0	94 (3.7)	10 (2.6)

*BPA* British Paediatric Association, *ICD* International Classification of Diseases, *ID* intellectual disability.

^a^Includes microcephaly (*n* = 12) and corpus callosum agenesis (*n* = 8).

^b^Includes microcephaly (*n* ≤ 5), corpus callosum agenesis (*n* = ≤5) and holoprosencephaly (*n* = ≤5).

^c^Does not include oral clefts, craniosynostosis and related syndromes, craniofacial microsomia and other contributing syndromes.

### Associations of ID with CFA

As depicted in Table 3, CFA was associated with an increased prevalence of ID (adjusted prevalence ratio [PR] 5.27, 95% CI 4.44, 6.25) as well as for mild and moderate and severe ID. By contributing diagnoses, association with ID was highest in children with CFM (adjusted PR 8.05, 95% CI 4.61, 14.07). The associations were higher in children with syndromic diagnosis of CFA and its subtypes than those with non-syndromic diagnosis.

**Table 3:** Association of Intellectual disability with craniofacial anomalies (comparison group: comparison cohort).

	Unadjusted PR (95% CI)			Adjusted^a^ PR (95% CI)		
	All ID	Mild/moderate ID	Severe ID	All ID	Mild/moderate ID	Severe ID
Craniofacial anomalies	5.64 (4.76, 6.69)	5.47 (4.53, 6.61)	11.31 (7.01, 18.24)	5.27 (4.44, 6.25)	5.13 (4.24, 6.19)	10.62 (6.59, 17.12)
Non-syndromic	1.50 (0.97,2.31)	1.72 (1.11, 2.65)	—	1.41 (0.92, 2.19)	1.62 (1.05, 2.51)	—
Syndromic	12.24 (10.28, 14.57)	11.72 (9.61, 14.30)	32.72 (20.42, 52.43)	11.22 (9.40, 13.40)	10.78 (8.82, 13.17)	30.69 (19.17, 49.14)
*Contributing diagnosis*						
Orofacial clefts	5.18 (4.20, 6.39)	5.10 (4.06, 6.43)	10.09 (5.58, 18.24)	4.79 (3.88, 5.92)	4.73 (3.76, 5.96)	9.47 (5.24, 17.11)
Non-syndromic	1.59 (0.98, 2.57)	1.82 (1.12, 2.95)	—	1.49 (0.92, 2.42)	1.69 (1.04, 2.75)	—
Syndromic	11.76 (9.44, 14.66)	11.37 (8.87, 14.58)	31.83 (17.76, 57.05)	10.64 (8.51, 13.31)	10.35 (8.04,13.31)	29.83 (16.64, 53.48)
Craniosynostosis	6.36 (4.54, 8.92)	5.96 (4.06, 8.73)	12.26 (4.62, 32.50)	6.09 (4.33, 8.58)	5.72 (3.90, 8.41)	11.49 (4.33, 30.49)
Non-syndromic	1.27 (0.48, 3.36)	1.46 (0.55, 3.85)	—	1.24 (0.47, 3.27)	1.43 (0.54, 3.77)	—
Syndromic	16.48 (11.89, 22.84)	15.53 (10.57, 22.81)	43.60 (16.78, 113.26)	15.66 (10.97, 21.51)	14.39 (9.75, 21.24)	39.94 (15.41, 103.54)
Craniofacial microsomia	8.45 (4.76, 15.00)	7. 97 (4.16, 15.28)	25.02 (6.39, 97.97)	8.05 (4.61, 14.07)	7.58 (4.05, 14.20)	24.11 (6.16, 94.37)

*PR* prevalence ratio, *ID* intellectual disability, *CI* confidence interval.

^a^Adjusted for sex, mother’s age, race, socioeconomic disadvantage, remoteness, parity, plurality, birth weight and gestational age.

### Associations of ID with CFA relative to other congenital anomalies

As presented in Table 4, while the prevalence of all ID among CFA was slightly higher than in those born with other congenital anomalies [adjusted PR 1.12, 95% CI 0.94, 1.33], this finding varied according to individual congenital anomalies. The prevalence of all ID among CFA was much lower than in individuals born with either nervous system, cardiovascular or chromosomal birth defects. However, the prevalence of all ID among CFA was higher than in those with gastrointestinal, urogenital and musculoskeletal system defects. These findings were similar for mild and moderate and severe ID.

**Table 4:** Association between intellectual disability and craniofacial anomalies (comparison group: children with congenital anomalies but not CFA).

Comparison group (*n*)	Unadjusted^a^ PR (95% CI) of craniofacial anomalies			Adjusted^a^ PR (95% CI) of craniofacial anomalies		
	All ID	Mild/moderate ID	Severe ID	All ID	Mild/moderate ID	Severe ID
Other congenital anomalies (28,362)	1.12 (0.95, 1.33)	1.16 (0.97, 1.40)	0.95 (0.59, 1.54)	1.12 (0.94, 1.33)	1.14 (0.94, 1.38)	1.08 (0.67, 1.76)
Nervous system (1290)	0.28 (0.24, 0.34)	0.32 (0.26, 0.39)	0.10 (0.06, 0.17)	0.30 (0.25, 0.37)	0.34 (0.27, 0.42)	0.12 (0.07, 0.20)
Cardiovascular (5168)	0.86 (0.72, 1.02)	0.84 (0.69, 1.02)	0.98 (0.58, 1.67)	0.89 (0.71, 1.04)	0.82 (0.67, 1.01)	1.14 (0.66, 1.96)
Respiratory (170)	0.94 (0.58, 1.52)	1.04 (0.60, 1.80)	0.68 (0.20, 2.29)	0.90 (0.53, 1.52)	0.94 (0.52, 1.69)	1.01 (0.24, 4.33)
Gastrointestinal (2006)	1.28 (1.02, 1.60)	1.35 (1.06, 1.73)	0.98 (0.53, 1.81)	1.36 (1.07, 1.72)	1.40 (1.08, 1.82)	1.19 (0.64, 2.24)
Musculoskeletal (6716)	1.30 (1.08, 1.56)	1.33 (1.09, 1.63)	1.10 (0.65, 1.85)	1.15 (0.95, 1.40)	1.17 (0.95, 1.45)	1.05 (0.62, 1.78)
Urogenital (9028)	1.81 (1.51, 2.18)	1.78 (1.46, 2.18)	2.54 (1.46, 4.45)	1.94 (1.60, 2.35)	1.88 (1.52, 2.32)	2.88 (1.64, 5.04)
Integument (2368)	2.60 (2.00, 3.38)	2.59 (1.95, 3.45)	3.73 (1.61, 8.62)	2.21 (1.68, 2.90)	2.17 (1.61, 2.92)	3.52 (1.52, 8.13)
Chromosomal (418)	0.17 (0.14, 0.21)	0.16 (0.13, 0.20)	0.08 (0.05, 0.15)	0.16 (0.13, 0.20)	0.15 (0.12, 0.18)	0.09 (0.05, 0.15)

*PR* prevalence ratio, *ID* intellectual disability, *CI* confidence interval.

^a^Adjusted for sex, mother’s age, race, socioeconomic disadvantage, remoteness, parity, plurality, birth weight and gestational age.

### Associations of ASD (with/without ID) with CFA

Based on 11 cases, the prevalence of ASD among children with CFA was more than twice as high as that of children without CFA (adjusted PR 2.11, 95% CI 1.16, 3.80) (Table 5). Similar to the results for ID, the prevalence of ASD among children with syndromic diagnosis of CFA was higher than those with non-syndromic diagnosis. On the other hand, the adjusted prevalence ratio of ASD among non-syndromic CS was marginally less than those with syndromic CS.

**Table 5:** Associations of autism spectrum disorder^a^ with craniofacial anomalies.

	Unadjusted PR (95% CI)	Adjusted^b^ PR (95% CI)
Craniofacial anomalies	2.28 (1.26, 4.11)	2.11 (1.16, 3.80)
Non-syndromic	1.01 (0.33, 3.12)	0.93 (0.30, 2.88)
Syndromic	4.32 (2.17, 5.59)	3.98 (2.00, 7.95)
*Contributing diagnosis*		
Orofacial clefts	1.73 (0.78, 3.84)	1.62 (0.73, 3.61)
Non-syndromic	—	—
Syndromic	4.92 (2.22, 10.88)	4.67 (2.11, 10.37)
Craniosynostosis	4.72 (1.98, 11.25)	4.01 (1.69, 9.51)
Non-syndromic	4.28 (1.39, 13.15)	3.67 (1.21, 11.18)
Syndromic	5.64 (1.43, 22.23)	4.72 (1.20, 18.60)
Craniofacial microsomia	—	—

*PR* prevalence ratio, *CI* confidence interval.

^a^With and without intellectual disability.

^b^Adjusted for sex, mother’s age, race, socioeconomic disadvantage, remoteness, parity, plurality, birth weight and gestational age.

### Age at diagnosis of ID and ASD among individuals with CFA

The median age at diagnosis of ID and ASD among children with CFA was between 3 and 4 years (ID: 3.5, IQR 1, 7; ASD: 4, IQR 2, 5) (Table 6). Most ID among children with CFA was identified by Disability Services Commission (DSC) (62.5%) and the remaining were only identified by the Education Department. The median age of diagnosis of ID among cases identified by DSC was much lower (4, IQR 2, 6) when compared to those only identified by the Department of Education WA (10.0, IQR 8, 13). ID was identified on average much later among children with non-syndromic oral clefts and CS than syndromic conditions.

**Table 6:** Age at diagnosis of intellectual disability and autism spectrum disorders among children born with craniofacial anomalies.

Craniofacial anomalies and subtypes (*n*)	Intellectual disability		Autism spectrum disorder	
	*n*	Age at diagnosis in years median (IQR)	*n*	Age at diagnosis in years median (IQR)
Craniofacial anomalies (1415)	134	3.5 (1, 7)	11	4 (2, 5)
Non-syndromic (878)	23	5 (4, 10)	≤5	4 (2, 10)
Syndromic (543)	111	3 (1, 7)	8	4 (2.5, 4.5)
*Contributing diagnosis*				
Orofacial clefts (1029)	89	3 (1, 7)	6	4 (3, 5)
Non-syndromic (673)	18	5.5 (4, 8)		—
Syndromic (361)	71	3 (1, 7)	6	4 (3, 5)
Craniosynostosis (303)	34	4 (2, 9)	≤5	4 (2, 4)
Non-syndromic (201)	≤5	10 (3, 17)	≤5	4 (2, 10)
Syndromic (101)	29	3.5 (1, 8)	≤5	2.5 (1, 4)
Craniofacial microsomia (76)	10	4.5 (3, 7)		—
*Contributing syndromes*				
Crouzon syndrome (15)	≤5	9 (5.5, 12.5)	0	—
CFM (Goldenhar syndrome) (61)	9	4 (3, 7)	0	—
Pierre Robin sequence (106)	16	7 (2, 9.5)	0	—

CFM Craniofacial microsomia

### Associations of ID and CFA by demographic, antenatal and perinatal factors

The relative differences in prevalence ratio of ID according to demographic, antenatal and perinatal factors are shown in Supplementary Table S4. It is well known that ID is much commoner in males than females.^25^ Thus 5634/257,132 (2.2%) of males compared with 2967/244,467 (1.2%) of females in those without CFA had ID. However, in the 1251 individuals with CFA considered for this analysis, 71/705 (10%) of males had ID compared with 50/546 (8.8%) of females. While the proportions of males with ID in both the comparison and CFA groups were higher than females, this relative proportional difference was almost twice as high for the comparison group [2.2% (males) vs 1.2% (females)] compared to the CFA group [10.0% (males) vs 8.8% (females)]. Hence the relative prevalence of ID with CFA in males was about two-fifths lower than that of females (adjusted relative prevalence ratio (RPR) 0.62, 95% CI 0.44, 0.87).

It is also known that paternal age is likely to be higher in individuals with ID.^46^ Thus 1743/ 65,129 (2.7%) of those without CFA and fathers >40 years had ID in this study compared with 6858/436,470 (1.6%) of those without CFA and fathers under 40 years. However, of the 1251 individuals with CFA considered for this analysis, 19/189 (10.1%) of those with fathers over 40 years had ID compared with 102/1062 (9.6%) of those with fathers under 40 years. Thus, the differential association was about 38% lesser among children with CFA born to older fathers than younger fathers (adjusted RPR 0.62, 95% CI 0.39, 0.97).

Thirdly it is widely known that ID is more prevalent among lower socioeconomic households.^47^ Hence, we observed the highest proportion of ID among the most disadvantaged quintile (Q5) for both the comparison group 3201/114,304 (2.8%) and the CFA 43/286 (15%) and. Furthermore, we observed the lowest proportion of ID among the least disadvantaged quintile (Q5) relative to other quintiles in the comparison cohort [0.9%; (810/86,047)] but not for the CFA group [10%; (21/210)]. Thus, the observed prevalence ratios of ID and CFA for socioeconomically disadvantaged households (Q1, Q2) were about half as those who were born into households with least socioeconomic disadvantage.

## Discussion

Our findings indicate a higher prevalence of all ID and ASD among children born with CFA relative to those born without CFA. While the prevalence of ID among CFA was higher than those born with other gastrointestinal, urogenital, musculoskeletal and integument anomalies, it was lower than those born with other nervous system and chromosomal abnormalities. Furthermore, we observed higher proportions of other nervous system anomalies and musculoskeletal among children with CFA and ID relative to those with other congenital anomalies and ID. The majority of ID observed in CFA was mild to moderate occurring mostly due to an unknown cause (especially non-syndromic oral clefts). We also observed higher levels of ID and ASD among children with syndromic conditions. We, however, observed no ID among children born with Muenke syndrome and Treacher Collins syndrome. The mean age of diagnosis for ID was higher for children born with either non-syndromic oral cleft or CS than those with syndromic conditions.

There are limited population-based studies on the association of either ID or ASD among children born with CFA. The use of total population data for the state of WA to determine this association was a strength of this study, as was the ability to link midwives and other data sets to explore the association of potential antenatal and perinatal factors with these disease conditions. Additionally, the multiple source ascertainment of both CFA and ID/ASD from multiple sources has maximised the ascertainment of these conditions in the population. These data are inclusive and thus reduce any chance of selection bias.^48^

However, the low case numbers for individual contributing diagnoses of CFA and diagnosed ASD might limit our ability to estimate with acceptable precision. Syndromic oral clefts and CS category comprises of a heterogeneous group of rare syndromes with different distinct (often genetic) etiologies which may confound analysis. Our selection criteria included all births surviving to 5 years of age as a result of which those children with CFA who were diagnosed early with ID (*n* = 7), especially severe ID (6/7), but died before their fifth birthday, were excluded. While these numbers were not substantial, our findings potentially underreport the prevalence of severe ID among CFA. It is also important to note that we did not consider the timing of craniofacial/neurosurgical interventions which could potentially influence neurocognitive outcomes.^49^ While we could not report the most recent findings regarding association due to the limited period of data access (1983–2010), our study has provided unique population data analysis not previously reported in the literature.

Among syndromic causes, the current study observed ID among children with Pfeiffer syndrome and Apert syndrome but did not identify any ID among children born with Treacher Collins syndrome, which is in line with findings from earlier research.^50–52^ However, in contrast to some earlier hospital-based studies,^51,53,54^ we identified ID in children diagnosed with Crouzon syndrome. We also observed no ID among our small cohort of Muenke syndrome (<5 cases) while the Australian Craniofacial Unit, South Australia identified one child with ID in their sample of five Muenke syndrome cases.^53^

In categories where birth defects involve an obvious structural or functional disturbance of the nervous system, there is a plausible explanation for impairment in intellectual function. For instance, in the case of syndromic synostosis (e.g. Apert, Crouzon and Pfeiffer syndrome) modern imaging has demonstrated multiple brain abnormalities which are mostly non-specific (e.g. ventromegaly) and some related to mechanical constraints of cerebral growth (e.g. Chiari I) and certain malformations (e.g. agenesis of corpus callosum).^55^ We also observed ID among around 20% of children with CFM, which was not observed in an earlier North American study wherein 121 adolescents assessed had normal intelligence.^17^ However, it must be acknowledged that direct comparisons with individual studies are limited by different assessment methods used to determine ID, age at ascertainment, the size of cohort evaluated and the scheduled timing of craniofacial/ neurosurgical intervention.

The proportion of ID among CFA in the current study was slightly higher than in children diagnosed with other gastrointestinal, urogenital, musculoskeletal and integument disorders but was lower than those diagnosed with chromosomal (including Down syndrome) and non-chromosomal nervous system anomalies similar to that observed in an earlier Western Australian cohort (1980–1999).^26^ We believe the higher prevalence of ID, especially severe ID, among CFA individuals relative to those with other gastrointestinal, urogenital, musculoskeletal and integument disorders could potentially be a result of the presence of additional nervous system anomalies (especially microcephaly, corpus collosum agenesis and holoprosencephaly) among those with CFA. However, it is imperative further studies utlising this population evidence are essential to explore causal pathways.

Despite different assessment methods of ID used by individual studies, most studies including our current study found a higher prevalence of ID among our study group relative to those without CFA.^16^ The majority of the identified cases had a mild to moderate ID, but we observed a far higher proportion of nervous system anomalies for children with severe ID which could explain the reason for the severity.

While the majority of ID identified in the current study was deemed to have an unknown cause, it is possible that some cases of ID ascertained through education providers may have a known cause, which is not available to the IDEA Database and which could contribute to under-ascertainment of syndromes with a known diagnosis of ID.^26^

The age at diagnosis of ID for non-syndromic CFA in our cohort was higher than for syndromic cases. These findings are not surprising considering all non-syndromic cases in our cohort presented with mild and moderate ID which may not be diagnosed until early school age when their difficulties in academic learning become apparent.^56^

We did not observe any notable difference in the associations of ID with CFA with respect to underlying demographic, antenatal and perinatal characteristics except for sex, paternal age and socioeconomic disadvantage. As explained, we found a lower differential association of ID and CFA among males because of the high proportion of ID in males overall. Similarly, we found a lower differential association of ID and CFA with older fathers (≥40 years) because of the higher prevalence of older fathers in children with ID.

While CFA have not previously shown any association with socioeconomic status,^29,57^ ID is more prevalent among low socioeconomic groups.^46,47^ We observed a decreasing trend in the prevalence of ID with decreasing level of socioeconomic disadvantage in the comparison but not the CFA cohort. Considering the small numbers of ID among the CFA cohort, the relative differential associations which we observed for different socioeconomic disadvantaged households in our study cohort potentially indicate a spurious association rather than a definitive relationship.

As with ID, syndromic craniofacial conditions presented with a higher prevalence of ASD similar to an earlier WA study, which investigated the prevalence of autism in children with birth defects.^27^ Although earlier case reports,^58^ reported a higher degree of autism among children with Goldenhar syndrome, our findings of no autism among 63 cases of CFM (Goldenhar syndrome) contradict these findings, much like an earlier Western Australian population-based study.^27^ Among potential reasons for differences, one could be related to lack of universal agreement on minimal diagnostic criteria for CFM.^38^

Interestingly, we observed a higher prevalence of ASD among children born with non-syndromic CS than in those without this birth defect. Previous studies had reported autistic tendencies among children born with trigonocephaly (metopic synostosis) which were reduced after timely neurosurgical interventions for trigonocephaly.^49,59^ We however believe our finding of higher ASD among children with non-syndromic CS is limited by the small absolute number of observed cases with autism.

Understanding the prevalence of ID and ASD among children with CFA is important in commissioning health services, which will eventually improve health outcomes. Findings of our study reinforce the importance of early identification of ID and ASD, and its underlying cause, through a combination of phenotypic and molecular genetic assessment. In the future, population data augmented by clinical research, international registries and improved rare diseases coding is key to better understanding neurocognitive outcomes when identified with CFA especially rare syndromic conditions.

## Conclusions

This study provides unique population-level data analysis indicating a higher prevalence of ID and ASD among children born with CFA especially with syndromic conditions relative to those born without CFA. Furthermore, the prevalence of ID among CFA was higher than those born with other gastrointestinal, urogenital, musculoskeletal and integument anomalies but lower than chromosomal and nervous system anomalies. Most of the ID identified in our cohort was mild to moderate in severity, among children with additional nervous system anomalies and a result of an unknown definitive cause. The presence of CFA among children with ID suggests an aetiology that warrants further investigations for possible genetic and/or environmental antecedents. Conversely, awareness of the extra-cranial and neurocognitive CFA phenotype facilitates early identification and intervention of ID and ASD among children with CFA, thereby improving life outcomes.

## Supplementary information

Supplemental file

Supplementary Table 2

Supplementary Table 3

Supplementary Table 4
