# Supplementary material for: Association between craniofacial anomalies, intellectual disability and autism spectrum disorder: Western Australian population-based study
Source: Pediatr Res. 2022 Mar 29;92(6):1795–804. doi: 10.1038/s41390-022-02024-9 (PMC9771801; doi:10.1038/s41390-022-02024-9)
Supplement: Supplementary file 1 — Supplemental file [file 41390_2022_2024_MOESM1_ESM.pdf]

Supplemental Figure S1: Summary of total number of individuals, and data extracted from each data source used in this study.

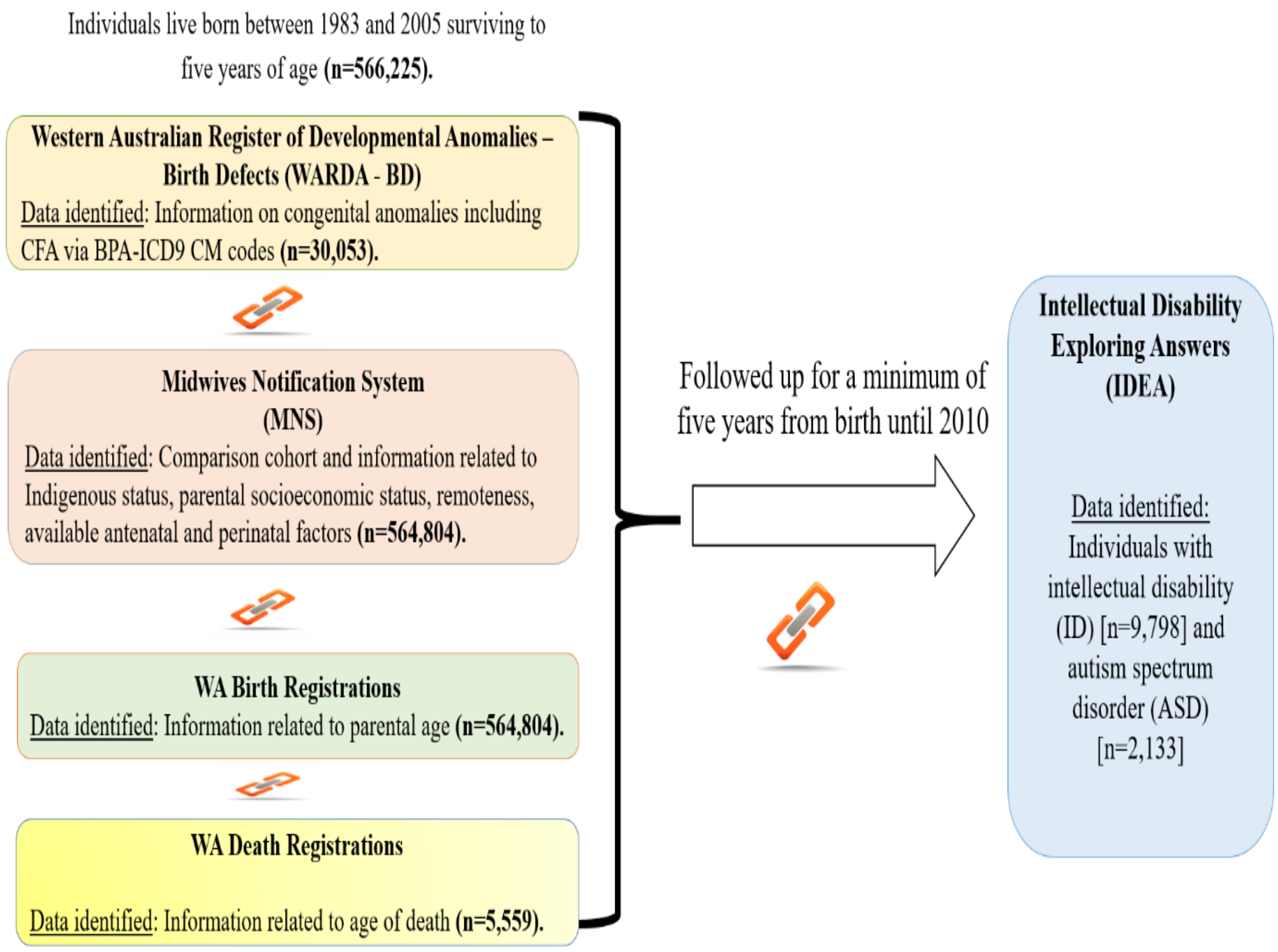

CFA- Craniofacial anomalies; BPA-ICD9CM – British Paediatric Association- International Classification of Diseases 9 Clinical Modification.

Supplemental Figure S2: Total number of cases considered for data analysis after excluding for invalid records and missing [socioeconomic disadvantage (IRSD) and remoteness (ARIA)] data.

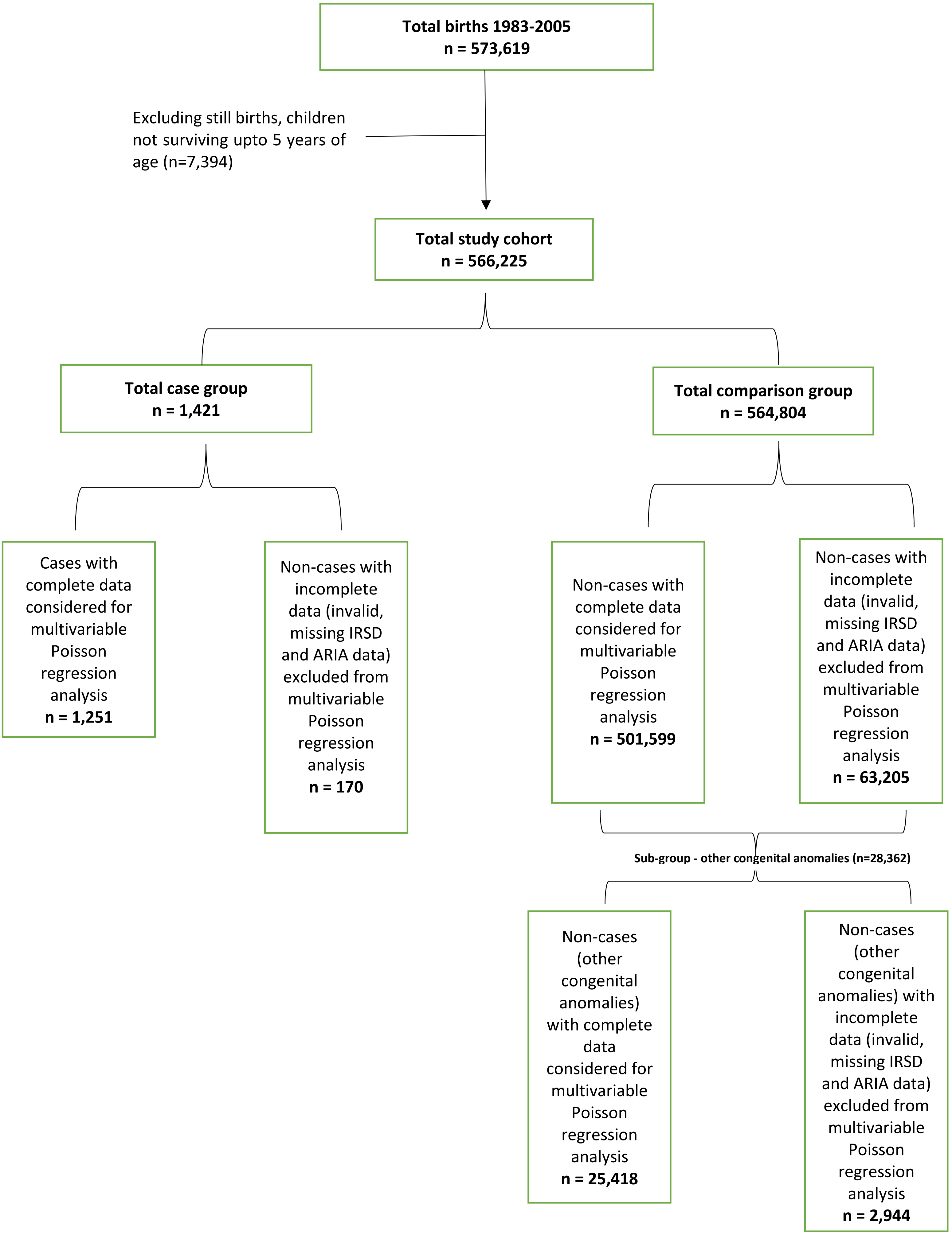

Supplemental Table S1 : ICD-9-CM and ICD-10-AM codes used to identify diabetes mellitus, epilepsy and hypertension from the MNS.

Mother medical condition	ICD-9-CM codes [a]	ICD-10-AM codes [b]
Diabetes Mellitus	250 (250.0 – 250.92);<br>648.0 – 648.84	E10.0 to E14.9
Epilepsy	345.0 – 345.91	G40.0 – G41.9
Hypertension	401.0 – 405.99; 416.00	I10 – I15.9; I27.0

ICD-9-CM: International Classification of Diseases – ninth revision – Clinical modification;

ICD-10-AM: International Classification of Diseases – tenth revision – Australian modification;

MNS: Midwives Notification System

[a] Used to identify data between 1980 and 1997

[b] Used to identify data between 1998 and 2010.
